# Structural characterization of scorpion peptides and their bactericidal activity against clinical isolates of multidrug-resistant bacteria

**DOI:** 10.1371/journal.pone.0222438

**Published:** 2019-11-11

**Authors:** Catherine Cesa-Luna, Jesús Muñoz-Rojas, Gloria Saab-Rincon, Antonino Baez, Yolanda Elizabeth Morales-García, Víctor Rivelino Juárez-González, Verónica Quintero-Hernández

**Affiliations:** 1 Ecology and Survival of Microorganisms Group (ESMG), Laboratorio de Ecología Molecular Microbiana (LEMM), Centro de Investigaciones en Ciencias Microbiológicas (CICM), Instituto de Ciencias (IC), Benemérita Universidad Autónoma de Puebla (BUAP), Puebla, Puebla, México; 2 Departamento de Ingeniería Celular y Biocatálisis, Instituto de Biotecnología, Universidad Nacional Autónoma de México, Cuernavaca, Morelos, México; 3 Licenciatura en Biotecnología, Facultad de Ciencias Biológicas, BUAP, Puebla, Puebla, México; 4 Departamento de Medicina Molecular y Bioprocesos, Instituto de Biotecnología, Universidad Nacional Autónoma de México, Cuernavaca, Mor., México; 5 CONACYT-ESMG, LEMM, CICM, IC, BUAP, Puebla, Puebla, México; University of Padova, Medical School, ITALY

## Abstract

Scorpion venom peptides represent a novel source of antimicrobial peptides (AMPs) with broad-spectrum activity. In this study, we determined the minimum bactericidal concentration (MBC) of three scorpion AMPs, Uy234, Uy17, and Uy192, which are found in the venomous glands of the *Urodacus yaschenkoi* scorpion, against the clinical isolates of multidrug-resistant (MDR) bacteria. In addition, we tested the activity of a consensus AMP designed in our laboratory based on some previously reported IsCT-type (cytotoxic linear peptide) AMPs with the aim of obtaining higher antimicrobial activity. All peptides tested showed high antimicrobial activity against MDR clinical isolates, with the highest activity against β-hemolytic *Streptococcus* strains. The hemolytic activity was determined against human red blood cells and was significantly lower than that of previously reported AMPs. The α-helical structure of the four AMPs was confirmed by circular dichroism (CD). These results suggest that the four peptides can be valuable tools for the design and development of AMPs for use in the inhibition of MDR pathogenic bacteria. A clear index of synergism and additivity was found for the combination of QnCs-BUAP + Uy234, which makes these peptides the most promising candidates against pathogenic bacteria.

## Introduction

Scorpions are arthropods belonging to the group of arachnids, which have lived on our planet for more than 400 million years. Currently, approximately 1500 species of scorpions have been described, and the study of their venoms has resulted in the discovery of a large arsenal of bioactive molecules [1]; among these molecules, toxins are distinguished by their ability to modulate the function of diverse ion channels and membrane receptors, causing the symptoms of poisoning [2]. Other interesting biomolecules that have been discovered in scorpion venoms are antimicrobial peptides (AMPs), which stand out for their broad-spectrum activity against Gram-positive and Gram-negative bacteria, fungi, yeast and protozoa. The most interesting AMPs from scorpion venoms are those falling out of Buthidae family, which do not present any risk to human health [3]; The venoms contain antimicrobial peptides (AMPs) involved in the innate immune response of venomous arthropods [4]. Scorpion AMPs also function as internal immune agents protecting the venom gland from infection and external immune agents cleaning saprophytic microbes from surface of scorpion body [5–7]. In addition, synergistic effects of antimicrobial scorpion peptide and neurotoxins against bacteria has been described, which provides further evidence in favor of multifunctionality of scorpion venom components [8]. Most of these antimicrobial peptides do not contain disulfide bridges in their structure, known as non-disulfide-bridged peptides (NDBP), and have been classified into several groups, such as scorpine-like, long, intermediate and short-chain AMPs [1,3].

Scorpine-like AMPs possess two structural and functional domains: a N-terminal α-helix (with cytolytic and/or antimicrobial activity like the insect defensins) and a tightly folded C-terminal region with a CSαβ motif, displaying K+ channel-blocking activity [9]. The first characterized scorpine was isolated from the venom of the scorpion *Pandinus imperator*. This protein is formed by 75 amino acids and has shown antimicrobial activity, mainly against *Bacillus subtilis*, although it also has an inhibitory effect on the ookinete and gamete stages of *Plasmodium berghei* [10].

Long-chain AMPs, such as hadrurin (isolated from Mexican scorpion *Hadrurus aztecus*), belong to the superfamily of peptides without disulfide bridges [11]. This 41-amino acid peptide forms helical structures and has antimicrobial activity against Gram-negative bacteria, such as *Salmonella typhi*, *Klebsiella pneumoniae*, and *Pseudomonas aeruginosa*. Other related peptides, such as Opistoporin-1 (*Opistophthalmus carinatus*) and Pandinin-1 (*Pandinus imperator*), both with 44 amino acids, have antimicrobial activity against Gram-positive and Gram-negative bacteria, respectively [12,13].

AMPs from the scorpion *Heterometrus spinifer* have been reported within the group of AMPs with an intermediate size peptide chain; one of these peptides is HsAp (29 amino acids), which has broad-spectrum antibacterial activity against both Gram-positive and Gram-negative bacteria, as well as antifungal activity [14]; however, HsAp has limited therapeutic use because it is highly hemolytic against human erythrocytes. Other peptides included in this classification are: Heterin-2 and pandinin-2. Heterin-2 is a venom peptide from the scorpion *Heterometrus spinifer* which contains 24 amino acid residues. Heterin-2 is able to inhibit the growth of Gram-positive bacteria with MICs from 5.6 μM to 30.0 μM [15]. Pandinin-2 is an α-helical polycationic antimicrobial peptide from venom of the scorpion *Pandinus imperator*. Pandinin-2 contains 24 amino acid residues and demonstrated high antimicrobial activity against a range of Gram-positive bacteria (2.4–5.2 μM), but was less active against Gram-negative bacteria (2.4–38.2 μM) [12].

Short-chain AMPs or IsCT-type peptides are short antimicrobial peptides derived from longer peptide precursors by post-translational processing of a signal peptide and a carboxy-terminal propeptide region that flank the mature peptide. The mature peptide contains commonly less than 20 amino acids, most of them hydrophobic.

Currently, 67 IsCT-type AMPs have been reported [1]. The first IsCT peptide was isolated from the venom of the scorpion *Opisthacanthus madagascariensis*; it was named in this way because the scorpion was found in Isalo (Is), Madagascar and the peptide displayed cytotoxic activity (CT). IsCT peptide presents antibacterial activity against Gram-negative and Gram-positive strains of bacteria and low hemolytic activity [16]. Other short peptides from Mexican scorpions have also been identified, *e*.*g*., VmCT1 and VmCT2 from the scorpion *Vaejovis mexicanus*. From the cDNA libraries made from the venom glands of this scorpion belonging to the Vaejovidae family, the sequences of these peptides were identified. The chemically synthesized peptides (with C-terminal amidation) showed potent antibacterial activity as well as low hemolytic activity [17].

The mechanism of action of these AMPs is unclear, but it has been proposed that these AMPs act in several stages [18]: (1) electrostatic interaction onto the membrane surface; (2) a peptide threshold concentration is reached before membrane rupture can occur, after which three main models have been proposed: I) the carpet model [19], the peptides remain parallel to the bilayer and cause an effect similar to detergent, II) the barrel stave pore model, the peptides are inserted perpendicularly into the bilayer and there is an auto-association that forms a pore that contains peptide-peptide interactions [20,21], and III) the toroidal model [22] indicates a mechanism of pore formation in which the lumen of the pores is coated with peptides and phospholipids in a less rigid association. Previous reports have shown that the amidation of the C-terminus of AMPs can stabilize the secondary structure (the secondary structure prediction analysis shows that these IsCT-type peptides can fold into an amphipathic-helix) and enhance the affinity of AMPs towards the membrane [23–25].

Recently, as a result of the transcriptomic analysis of the venomous glands of the *Urodacus yaschenkoi* scorpion, the existence of a large number of peptides with potential antimicrobial activity (mainly NDBP type) was discovered. The antimicrobial activity of several of these peptides (chemically synthesized) has been evaluated [26–28], e.g., the UyCT3 peptide with an amidated C-terminal showed strong antimicrobial activity, while the non-amidated UyCT3 peptide failed to exhibit any antimicrobial activity, confirming the importance of the amidation of the C-terminus [16]. The development of new technology platforms for peptide synthesis and modification with a low cost makes peptide systems valuable tools for the design of new therapeutic weapons against multidrug-resistant (MDR) strains.

In this study, the bactericidal activities of three AMPs derived from *Urodacus yaschenkoi* scorpion venom and one AMP designed from a consensus sequence were evaluated against MDR bacterial strains (obtained from clinical isolates). In addition, the peptide hemolytic activities against human erythrocytes were evaluated, and their structures were determined by circular dichroism (CD). The four peptides were found to adopt an α-helix structure, showed great bactericidal activity against MDR clinical isolates, and had remarkably low hemolytic activity. Taken together, these results suggest that our peptides have broad-spectrum antibacterial activity and could be used for the development of new antimicrobial drugs.

## Materials and methods

### Ethics statement

Human red blood cells (hRBCs) were collected from the healthy donor Catherine Cesa-Luna, who is the author of this manuscript and gave her verbal consent for phlebotomy. The hRBCs-related experiment was approved by the Ethics Committee of the Universidad Nacional Autónoma de México and the Benemérita Universidad Autónoma de Puebla.

### Strains

The following Gram-positive and Gram-negative strains were used in this work: *Escherichia coli* (ATCC^®^ 25922^TM^), *Staphylococcus aureus* subsp. *aureus* (ATCC^®^ 25923^TM)^ and *Klebsiella pneumoniae* subsp. *pneumoniae* (ATCC^®^ 13883^TM^); *Burkholderia cepacia* and *Paraburkholderia silvatlantica* (isolated from sugarcane) [29]; and three MDR clinical isolates: *Klebsiella* sp. KP8, *Streptococcus* sp. SP10, and *Streptococcus* sp. ST9, obtained from ISSSTEP (Instituto de Seguridad y Servicios Sociales de los Trabajadores al Servicio de los Poderes del Estado de Puebla) Hospital, Puebla, México (identified with the API 20E system).

### The chemical synthesis and purification of peptides

**Fig 1:**
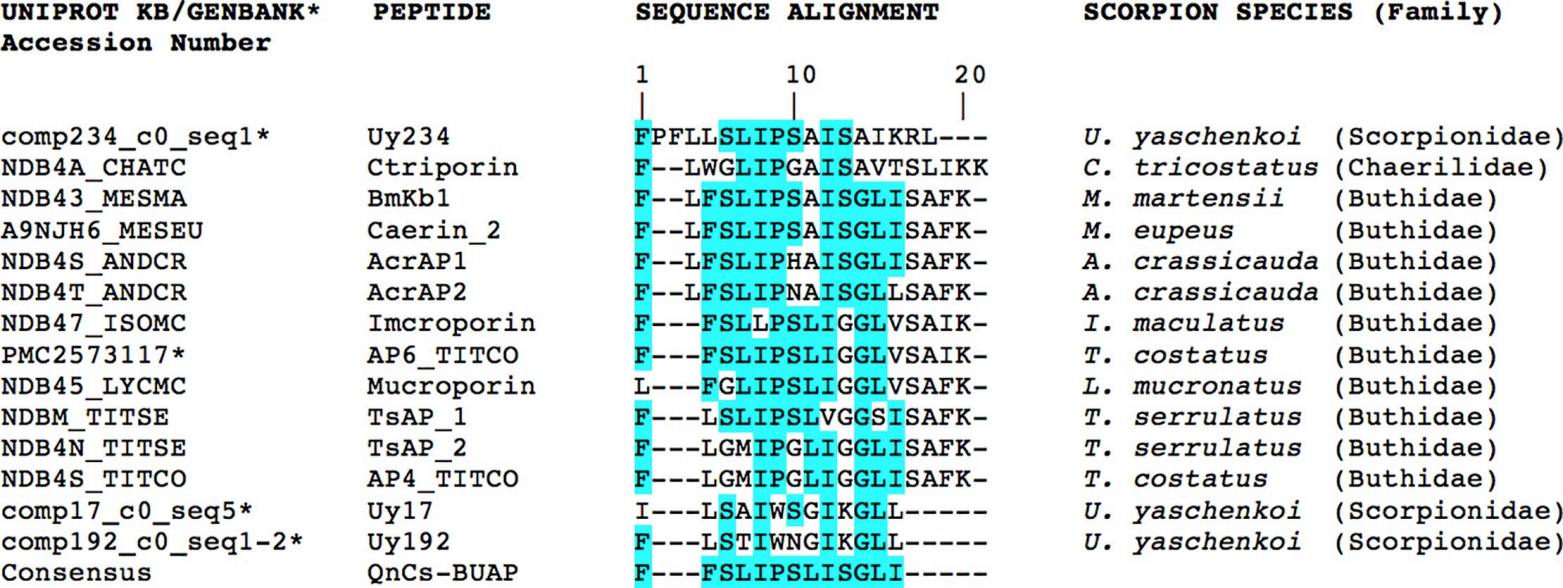
Sequence alignment of the AMPs that were used to obtain the consensus peptide QnCs-BUAP. The alignment was performed using the T-COFFEE program (http://tcoffee.crg.cat/apps/tcoffee/do:regular). *U = Urodacus*; *C = Chaerilus*; M = *Mesobuthus*; A = *Androctonus*; I = *Isometrus*; T = *Tityus*; L = *Lychas*.

The sequences and partial characterization of the three AMPs used in this study (Uy234, Uy17 and Uy192) were previously reported and were obtained by the transcriptomic analysis of mRNA from the venomous glands of the scorpion *Urodacus yaschenkoi* [26,28]. A fourth sequence (consensus peptide: QnCs-BUAP) was designed in our laboratory, based on the most conserved amino acids of some AMPs of the IsCT type from scorpion venoms that had been reported until 2016 by using the T-COFFEE program (http://tcoffee.crg.cat/apps/tcoffee/do:regular) (Fig 1). The AMPs from Fig 1 were selected because they are classified as short-chain non-cysteine-containing AMPs [30], which is the same as the peptides Uy234, Uy17, and Uy192. We also selected those peptides because of they had higher percentage of identity with the *Urodacus yashenkoi* peptides. A final consideration in choosing those AMPs from scorpion venoms was that the peptides should belong to both the Buthidae family (dangerous for humans) and non-Buthidae family (scorpion families different to Buthidae which are not dangerous for humans) to have greater diversity in the alignment and an optimal consensus sequence.

For the experimental evaluations, peptides were synthesized by Biomatik (https://www.biomatik.com/) (Ontario, CA) with an amidated C-terminus and were purified by HPLC using a C18 analytic column. The linear gradient used was from solvent A (0.1% trifluoroacetic acid in 100% acetonitrile) to solvent B (0.1% trifluoroacetic in 100% water) with a flow rate of 1.0 ml/min, runs for 30 min (the HPLC gradient used for each peptide is reported in S2–S5 Figs) with a purity of > 95%. The molecular masses were confirmed by mass spectrometry (S6–S9 Figs). The physicochemical properties are shown in Table 1.

**Table 1:** Physicochemical properties of the antimicrobial peptides used in this study.

Peptide	Amino acid sequence	Molecular mass, theoretical (Da)	Molecular mass, observed (Da)	Length (aa)	Net charge	GRAVY^1^
Uy234	FPFLLSLIPSAISAIKRL-NH2	1985.46	1985.51	18	+3	1.328
Uy17	ILSAIWSGIKGLL-NH2	1369.69	1369.73	13	+2	1.500
Uy192	FLSTIWNGIKGLL-NH2	1460.76	1460.80	13	+2	0.969
QnCs-BUAP	FFSLIPSLISGLI-NH2	1405.72	1405.76	13	+1	2.008

^1^ Grand average of hydropathy.

### Antimicrobial assays and synergistic antimicrobial effect

The microdilution method was performed according to Li *et al*., with the slight modifications indicated below, to determine the minimum bactericidal concentration (MBC) [31]. Synthesized peptides (lyophilized) were resuspended according to their solubility test (Biomatik, personal communication) and the highest concentrations selected for antimicrobial assays were: 380 μM (peptide Uy234), 372.5 μM (peptide Uy17), 339.3 μM (peptide Uy192) and 353.1 μM (peptide QnCs-BUAP). Afterwards, samples were added to 96-well ELISA plates and doble diluted (1:2) in Luria-Bertani (LB) using a multichannel pipette.

Previously, bacterial strains were cultured in LB medium and diluted to 10^5^−10^6^ CFU/ml. A total of 195 μl of peptide dilution and 5 μl of bacterial suspension were incubated for 16–18 h at 30°C and 150 rpm. The turbidity of each bacterial solution in the microplate was read at 620 nm in an ELISA plate reader and the bacterial growth was determined. Afterwards, to confirm the bactericidal activity (bacterial killing), a replicator was used to stamp all bacterial samples recovered from the wells onto LB agar plates. The Petri plates were incubated for 24 h at 30°C. A sterility control (without bacteria) and a control for the bacterial growth (without peptide) were included. The MBC for these bacteria was estimated as the lowest peptide concentration that showed bacterial killing on agar plates. To increase the bactericidal activity different peptide mixtures were performed, and the synergistic antimicrobial effect was evaluated. The combinations were Uy234 + QnCs-BUAP; Uy17 + QnCs-BUAP; and Uy192 + QnCs-BUAP. The bactericidal analysis was performed in a similar way to the antimicrobial assays, above mentioned, except that all peptides were adjusted to a final concentration of 300 μM. The three mixtures were added to 96-well ELISA plates with a final volume of 200 μl, containing 195 μl of each peptide mixture and 5 μl of the bacterial suspension. All antimicrobial assays were performed in duplicate. The average values and standard deviation were reported.

### Fractional inhibitory concentration (FIC)

Synergy data were analyzed according to Almaaytah *et al*. [32] by using the FIC index, which is defined as the ratio of the MBC for the peptide combination divided by the MBC of the single peptide. The equation is represented as follows:

$$FIC\;index=\frac{MBC\;of\;drug\;X\;in\;combination}{MBC\;of\;drug\;X\;alone}+\frac{MBC\;of\;drug\;Y\;in\;combination}{MBC\;of\;drug\;Y\;alone}$$

The FIC indices were interpreted as follows: ≤ 0.5 = synergism; 0.5–1 = additivity; 1–4 = indifference; and > 4 = antagonism.

### Hemolytic assays

The collected hRBCs were washed several times at 5000 rpm for 5 minutes in 1X PBS at pH 7.4 and were counted in a hemocytometer to adjust the cell concentration to 4.4 x 10^6^ cells/ml. The cell suspensions were incubated for 1 h at 37°C with different concentrations of each peptide. Ten percent Triton X-100 was used as a positive control (100% lysis), and 1X PBS was used as a negative control. After incubation, the samples were centrifuged for 5 minutes at 5000 rpm, and the supernatants were added to 96-well ELISA plates to measure the absorbance at 570 nm in an ELISA plate reader. The relative absorbance compared to Triton X-100 was defined the percentage of hemolysis [27] and the HC_25_ values, which represent the concentrations of peptide at which 25% hemolysis was observed, were determined. All assays were performed in triplicate.

### Secondary structure analysis and circular dichroism spectra

The secondary structure was predicted by using the online program NetWheels: Peptides Helical Wheel and Net projections maker (http://lbqp.unb.br/NetWheels/).

Peptide solutions were prepared with concentrations varying from 500–730 μM as follows: peptide Uy234 and Uy17 were dissolved in water; and Uy192 and QnCs-BUAP were dissolved in acetonitrile:H_2_O (1:4). Afterwards, the peptide solutions were filtered through 0.22 μm nylon filters. The far-UV CD spectra were recorded from 190 to 260 nm using a Jasco J-715 CD spectropolarimeter (JASCO Analytical Instruments) equipped with a Peltier temperature-controlled cell holder (PTC-4235, JASCO) in a 0.1 cm path-length quartz cell. Three spectra were averaged to reduce noise. Spectra were acquired every 1 nm, with eight seconds average time per point and a 1 nm bandpass.

The secondary structure was estimated from the CD spectra using the server dichroweb and the algorithm CDSSTR with the reference dataset [33].

## Results

### Antimicrobial assays

The antimicrobial activity of three peptides derived from *U*. *yaschenkoi* venom and one designed peptide were evaluated against Gram-positive and Gram-negative bacteria. The results are summarized in Table 2.

**Table 2:** Minimum bactericidal concentration of the antimicrobial peptides.

Bacterial strains	MBC ± standard error (μM)			
	Uy234	Uy17	Uy192	QnCs-BUAP
1. *Escherichia coli* ATCC 25922	190	186.2	> 339.3	> 353.1
2. *Staphylococcus aureus* ATCC 25923	29.6 ± 25	23.2	42.4	> 353.1
3. *Klebsiella pneumoniae* subsp. *pneumoniae* ATCC 13883	190	372.5	169.6	> 353.1
4. *Klebsiella* sp. KP (clinical isolate)	190	186.2	> 339.3	> 353.1
5. *Burkholderia cepacia*	> 380	> 372.5	> 339.3	> 353.1
6. *Paraburkholderia silvatlantica*	95	23.2	10.6	353.1
7. *Streptococcus* sp. SP10 (clinical isolate)	2.9	23.2	10.6	33.1 ± 16
8. *Streptococcus* sp. ST9 (clinical isolate)	5.9	11.6	15.9 ± 7	88.2

**Fig 2:**
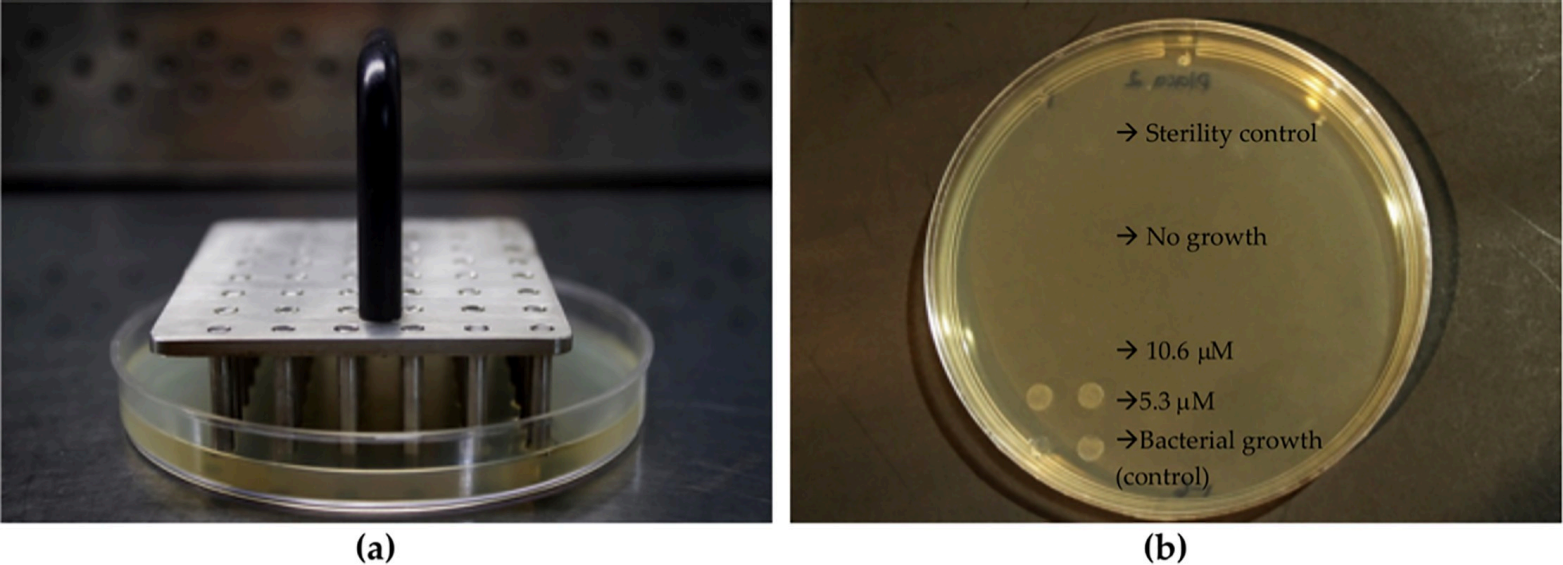
Representation of the antimicrobial assay. (A) Bacterial samples were stamped onto LB agar plates using a replicator. (B) Example of a plate after incubation, showing the bacterial killing of *Streptococcus* sp. SP10 (clinical isolate) with peptide Uy192 at 10.6 μM. Bacterial growth was observed at the lowest concentration tested (5.3 μM) and when the peptide was not present (negative control).

*Staphylococcus aureus* ATCC 25923, *Paraburkholderia silvatlantica*, *Streptococcus* sp. SP10, and *Streptococcus* sp. ST9 were the most sensitive to treatment with Uy234, Uy17, and Uy192. Uy234 showed the highest inhibitory activity against *Streptococcus* sp. SP10 and *Streptococcus* sp. ST9 (clinical isolates), with MBCs of 2.9 μM and 5.9 μM, respectively. The designed peptide, QnCs-BUAP, showed the least antimicrobial activity, and *Escherichia coli* ATCC 25922, *Staphylococcus aureus* ATCC 25923, *Klebsiella pneumoniae* subsp. *pneumoniae* ATCC 13883, *Burkholderia cepacia* and *Klebsiella* sp. KP8 (clinical isolate) were resistant to the highest concentration tested (353.1 μM); however, the consensus peptide showed great inhibitory activity against the MDR clinical isolates of *Streptococcus*. *Burkholderia cepacia* was resistant to the 4 peptides evaluated. The bactericidal activity was evaluated as indicated in the materials and methods, and the MBC of each peptide was determined at the micromolar concentration (Fig 2).

### Antimicrobial synergy assay

To quantify the interactions between the peptides that were tested, the consensus QnCs-BUAP peptide was combined with the Uy234, Uy17, and Uy192 peptides, and their antimicrobial synergy was evaluated (Table 3). When the Uy234 and QnCs-BUAP peptides were combined, the bactericidal activity was increased, suggesting a synergistic or additive effect. Hence, this peptide mixture showed antimicrobial activity against *Burkholderia cepacia*, which was resistant to single peptide exposure. The MBC of QnCs-BUAP combined with Uy234, Uy17, or Uy192, determined against *Escherichia coli* ATCC 25922, *Paraburkholderia silvatlantica*, and *Streptococcus* sp. ST9 was > 2-fold better compared to that observed for each of the single peptides, resulting in values that ranged from 2.34 to 75 μM. For *Staphylococcus aureus* ATCC 25923, there was no significant difference between the combination and single treatments.

**Table 3:** MBC for peptide combinations.

Bacterial strains	MBC ± standard error (μM)		
	Uy234 + QnCs-BUAP	Uy17 + QnCs-BUAP	Uy192 + QnCs-BUAP
1. *Escherichia coli* ATCC 25922	75	300	300
2. *Staphylococcus aureus* ATCC 25923	37.5	150	300
3. *Klebsiella pneumoniae* subsp. *pneumoniae* ATCC 13883	150	300	300
4. *Klebsiella* sp. KP8 (clinical isolate)	150	300	300
5. *Burkholderia cepacia*	150	> 300	300
6. *Paraburkholderia silvatlantica*	28.12 ± 13.26	37.5	14.06 ± 6.63
7. *Streptococcus* sp. SP10 (clinical isolate)	2.34	9.37	7.025 ± 3.32
8. *Streptococcus* sp. ST9 (clinical isolate)	2.34	9.37	3.51 ± 1.65

The mixture of the Uy17 and QnCs-BUAP peptides showed strong bactericidal activity against *Streptococcus* sp. SP10, with an MBC of 9.37 μM, while *Burkholderia cepacia* remained resistant (Table 3).

The synergistic activity of Uy192 and QnCs-BUAP was remarkable since QnCs-BUAP alone was unable to inhibit the growth of the *Escherichia coli* ATCC 25922, *Staphylococcus aureus* ATCC 25923, *Klebsiella pneumoniae* subsp. *pneumoniae* ATCC 13883, *Klebsiella* sp. KP8 (clinical isolate), and *Burkholderia cepacia* strains (Table 2). In addition, the bactericidal activity of this combination was increased more than 4-fold against *Paraburkholderia silvatlantica*, *Streptococcus* sp. SP10, and *Streptococcus* sp. ST9 (Table 3) compared with that of the single peptide activity (Table 2).

To further describe the synergistic behavior of peptide combinations, the fractional inhibitory concentration (FIC) index was calculated. The interpretation of the results is given in Table 4. As suggested by the results described above, peptide combinations with QnCs-BUAP showed synergistic or additive effects against all bacteria tested except for *Staphylococcus aureus* ATCC 25923. For *Staphylococcus aureus* ATCC 25923, the results showed indifferent or antagonistic effects, even though this strain was resistant to the QnCs-BUAP peptide. A significant result of this assay was the synergistic effects of the three peptide combinations on *Burkholderia cepacia*, even though *B*. *cepacia* was resistant to the single peptides (Table 4).

**Table 4:** Fractional inhibitory concentration (FIC) index values for the peptide combinations against the tested bacterial strains.

Microorganism	FIC index			Interpretation		
	Uy234 + QnCs- BUAP	Uy17 + QnCs- BUAP	Uy192 + QnCs- BUAP	Uy234 + QnCs- BUAP	Uy17 + QnCs- BUAP	Uy192 + QnCs- BUAP
1. *Escherichia coli* ATCC 25922	0.39	1.61	1	S	I	Ad
2. *Staphylococcus aureus* ATCC 25923	1.27	6.47	9.08	I	A	A
3. *Klebsiella pneumoniae* subsp. *pneumoniae* ATCC 13883	0.79	0.81	2.77	Ad	Ad	I
4. *Klebsiella* sp. KP8 (clinical isolate)	0.79	1.61	1	Ad	I	Ad
5. *Burkholderia cepacia*	0	0	0	S	S	S
6. *Paraburkholderia silvatlantica*	0.38	1.72	1.70	S	I	I
7. *Streptococcus* sp. SP10 (clinical isolate).	0.88	0.69	1.41	Ad	Ad	I
8. *Streptococcus* sp. ST9 (clinical isolate).	0.42	0.91	0.60	S	Ad	Ad

S: Synergism, Ad: Additivity, I: Indifference, A: Antagonism

### Hemolytic assays

**Fig 3:**
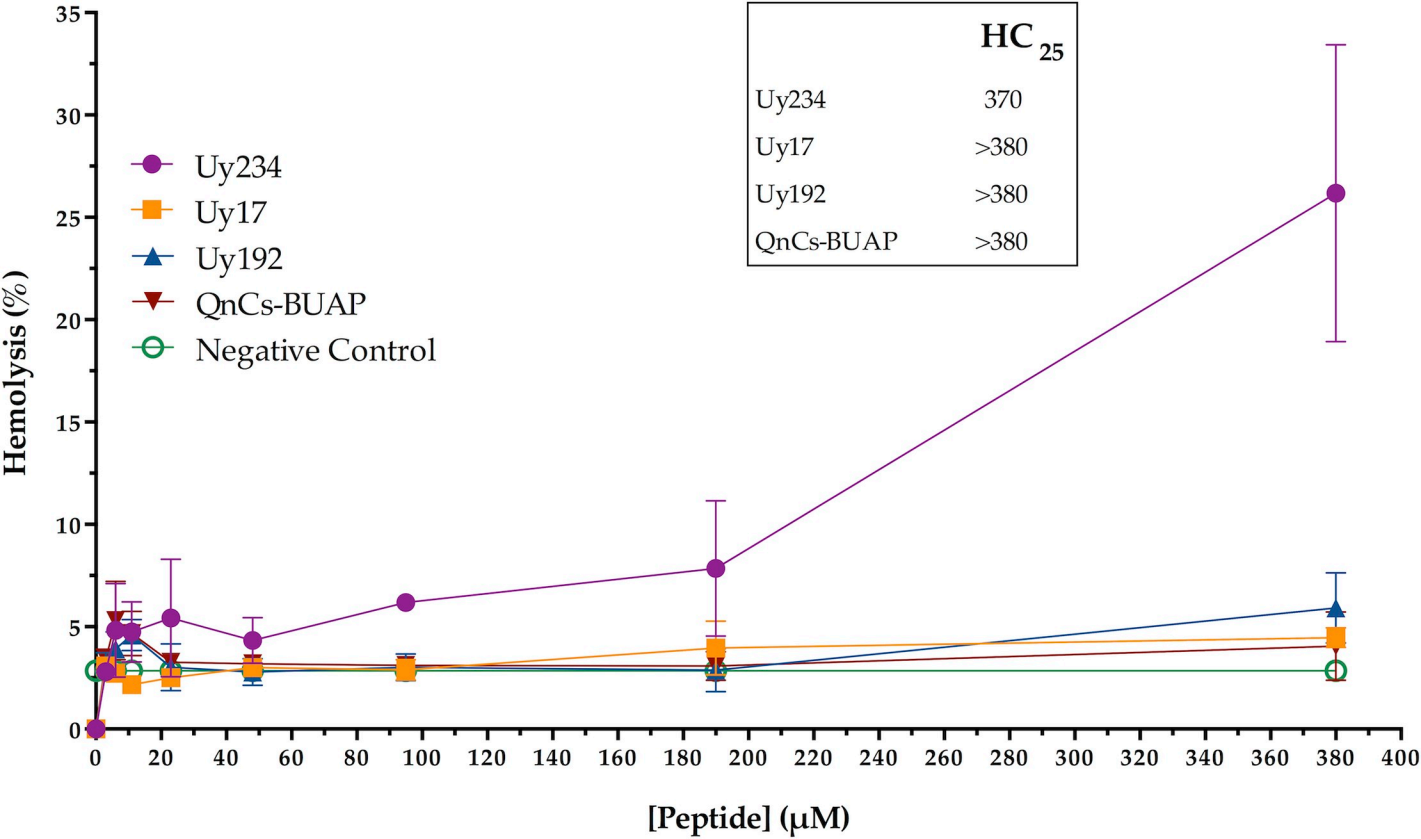
Hemolytic activity of the four peptides studied. Experiments were performed in triplicate by incubating human erythrocytes with different peptide concentrations (see Materials and Methods). The experimental values were normalized to the results for 1X PBS (0% hemolysis) and 10% Triton X-100 (100% hemolysis). Analysis was performed using GraphPad Prism Version 6.0 for Mac. The hemolytic concentration (HC_25_) obtained for each peptide is indicated in the box inside the figure.

The hemolytic activity was tested by co-incubating human erythrocytes with each peptide at different concentrations (Fig 3). Uy17, Uy192 and QnCs-BUAP were the least hemolytic peptides (activity lower than 6%), even at the highest concentrations tested. The Uy234 peptide showed 26.18% hemolytic activity at the highest concentration evaluated (380 μM).

When evaluated the hemolytic concentration (HC_25_) we could observe that peptide Uy234 required > 370 μM to achieve the HC_25_, while peptides Uy17, Uy192, and QnCs-BUAP required > 380 μM to achieve the same hemolytic concentration (see Fig 3).

### Secondary structure analysis and circular dichroism spectra

**Fig 4:**
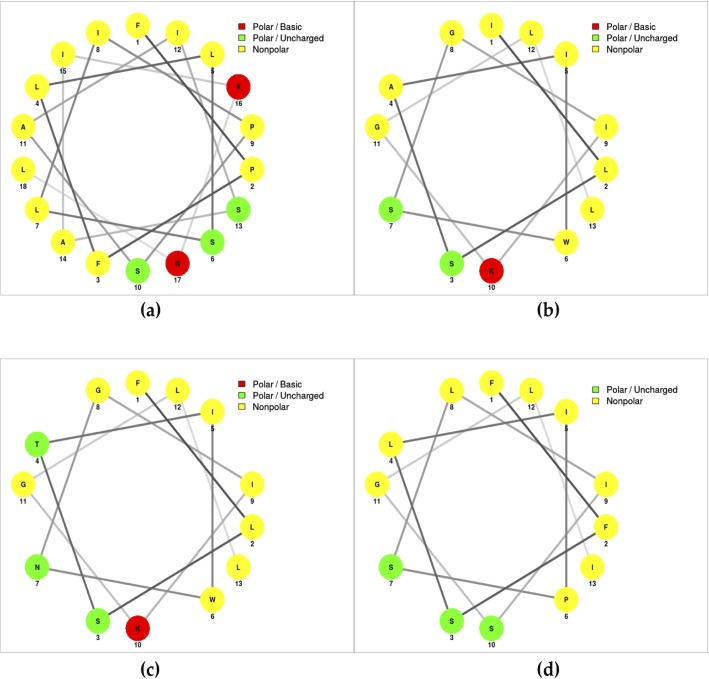
Representation of the helical wheel and net projections of the four peptides tested. a) Uy234 peptide: FPFLLSLIPSAISAIKRL; b) Uy17 peptide: ILSAIWSGIKGLL; c) Uy192 peptide: FLSTIWNGIKGLL; and d) QnCs-BUAP peptide: FFSLIPSLISGLI.

The secondary structure was predicted by using the online program NetWheels: Peptides Helical Wheel and Net projections maker (http://lbqp.unb.br/NetWheels/), which indicated that all peptides were compatible with a typical α-helix with a hydrophilic and hydrophobic face (Fig 4).

The CD spectra showed that the peptides were mainly disordered, with peptides Uy234 and QnCs-BUAP showing a higher signal at approximately 220 nm, indicative of some α-helix structure formation. The secondary structure content estimated from the CD spectra with the CDSTTR program from the server Dichroweb [34] is shown in Table 5.

**Table 5:** Estimation of the secondary structure elements of the AMPs used in this work.

%	Helix 1	Helix 2	Strand 1	Strand 2	Turns	Unordered	Total
1. Uy234	0.05	0.1	0.21	0.1	0.26	0.27	0.99
2. Uy17	0.03	0.08	0.3	0.1	0.19	0.29	0.99
3. Uy192	0.02	0.1	0.25	0.11	0.23	0.28	0.99
4. QnCs-BUAP	0.04	0.07	0.23	0.11	0.25	0.31	1.01
5. Uy192 (dilution)	0.02	0.09	0.2	0.11	0.26	0.32	1

**Fig 5:**
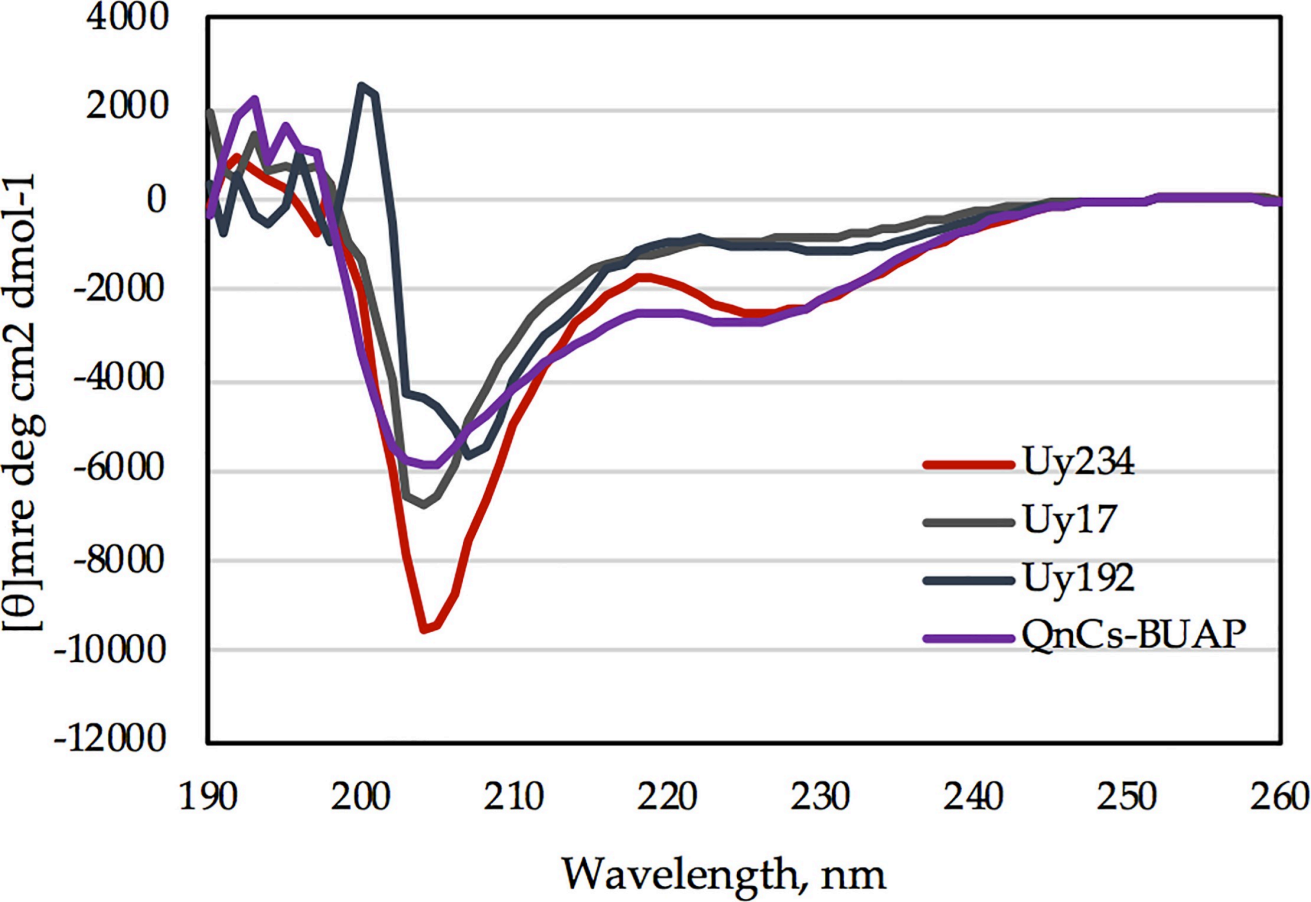
Far-UV CD spectra of the four peptides. The peptides were analyzed at concentrations varying between 500 and 730 μM.

As suggested by the Helical Wheel Projections, the peptides had the potential to form amphipathic helices, and the hydrophobic face of these helices would need to be stabilized to avoid aggregation, which can be accomplished through dimerization and the formation of a sort of small coiled-coil. We decided to dilute one of these peptides (Uy192) from 684 μM to 228 μM and observed that the minimum peak observed for this peptide moved from 208 nm to 198 nm (S1 Fig), indicating a concentration-dependent conformational change. These structures were confirmed by the CD analysis shown in Fig 5.

## Discussion

The rise of MDR bacteria represents a major public health problem worldwide [35]; consequently, global research is focused on the development of new therapeutic agents. Scorpion venoms represent an immense source of bioactive peptides, including toxins that affect the sodium channels of mammals or insects and AMPs [36–38]. However, only a few scorpion peptides with antimicrobial activity have been characterized and evaluated against MDR bacterial strains [1,9,39,40]. Among NDBPs with antimicrobial activity, scorpion venoms contain peptides such as Opistoporin, Pandinin or IsCT types [12,16,41].

*Urodacus yaschenkoi* is an Australian scorpion that has been characterized and contains an arsenal of biomolecules with diverse functions, including AMPs. Previous studies have reported that three of these peptides (UyCT1, UyCT3 and UyCT5) have been effective in inhibiting a broad spectrum of bacteria [27], including MDR pathogens such as *Acinetobacter baumannii* [42], and these peptides required the amidation of the C-terminal residue to be active.

In this work, we evaluated the structure, hemolytic and bactericidal activity of four synthetic AMPs, Uy234, Uy17, Uy192 and QnCs-BUAP, which is a completely novel consensus peptide. All peptides in this study were amidated at the C-terminal residue to enhance their solubility and potency [43]. To determine the antimicrobial activity, the minimal bactericidal concentration (MBC) of each peptide was determined against clinical isolates, two sugarcane isolates and three reference strains (ATCC). The clinical isolates included *Klebsiella* and β-hemolytic *Streptococcus*, which were obtained from pharyngeal exudates of patients with diverse illnesses and were MDR. *Klebsiella* sp. KP8 was resistant to erythromycin, ampicillin, penicillin, dicloxacillin and gentamicin, while *Streptococcus* sp. ST9 was resistant to ampicillin, netilmicin, enoxacin, penicillin, dicloxacillin, amikacin and gentamicin. To ensure that the antimicrobial activity determined by optical density was accurate, the bacterial growth of each strain was quantified by the “massive stamping drop plate” method [44], adjusting the initial bacterial cell concentration to 10^5^−10^6^ UFC/ml.

In contrast to most scorpion AMP studies in which the minimum inhibitory concentration (MIC) is the only parameter determined [17,38,39,45–48], in this work, the structure, hemolytic and bactericidal activity of the four AMPs was determined. The peptide Uy234 was the most effective, displaying the lowest MBC. *Streptococcus* sp. SP10 and *Streptococcus* sp. ST9 were the most sensitive strains, with MBCs of 2.9 μM and 5.9 μM, respectively. The remaining strains were also sensitive, but at higher concentrations of peptide. *Burkholderia cepacia* was the only strain resistant to the peptide Uy234, even at the highest concentration (380 μM).

Peptide Uy17 was also effective in inhibiting *Streptococcus* sp. SP10 and *Streptococcus* sp. ST9 at low concentrations (23.2 μM and 11.6 μM, respectively); furthermore, it was the only peptide able to inhibit the growth of *Staphylococcus aureus* ATCC 25923 at low micromolar concentrations (23.2 μM). The Gram-negative strain *Klebsiella pneumoniae* subsp. *pneumoniae* ATCC 13883 was sensitive only to the highest concentration of Uy17 tested (372.5 μM). On the other hand, the Uy192 peptide showed a low MBC for *Streptococcus* sp. SP10 and *Streptococcus* sp. ST9 (10.6 μM and 15.9 μM, respectively) was the most effective at inhibiting *Paraburkholderia silvatlantica*, with an MBC of 10.6 μM.

Few studies have revealed the efficacy of scorpion AMPs designed from the consensus sequences of highly conserved amino acids, which represents a very interesting strategy to boost antimicrobial activity. Among these sequences is the Pepcon consensus, which has broad-spectrum antimicrobial activity at very low micromolar concentrations against MDR strains such as *Acinetobacter baumannii* (20 μM) and methicillin-resistant *Staphylococcus aureus* (5 μM) [49]. Here, we designed a consensus peptide, QnCs-BUAP, that was able to completely inhibit the growth of *Streptococcus* sp. SP10 (33.1 μM) and *Streptococcus* sp. ST9 (88.2 μM).

*The Klebsiella* strains (ATCC 13883 and the clinical isolate) were resistant to even the highest concentrations that were tested for the peptides Uy192 and QnCs-BUAP (339.3 μM and 353.1 μM, respectively). This result is probably due to the presence of capsule and exopolysaccharide production, which are frequently responsible for resistance to diverse antibiotics [50]. Moreover, the presence of lipopolysaccharides rich in "highly acidic lipids" in the outer membrane of Gram-negative bacteria could influence the negative charge of the membrane, affecting the electrostatic binding of AMP to the cell surface [49].

The MIC values of scorpion venom antimicrobial peptides against Gram-positive and Gram-negative bacteria have been previously classified [30], as follows: Good activity (<10 μM), moderate activity (10–30 μM) and weak activity (> 30 μM). According to the results of Table 2 for *Streptococcus*, the peptide Uy234 reported in this study can be classified as good antimicrobial (activity <10 μM); other peptides that act at this concentration are: VpAmp1.0, VpAmp1.1 and VpAmp2.1 from *Vaejovis punctatus* [45]; VmCT1 from *Vaejovis mexicanus* and its analogs [5]; and UyCT5 from *Urodacus yaschenkoi* [51].

Peptides Uy17 and Uy192 can be considered with moderate activity (10–30 μM) against *Streptococcus* (see Table 2). Other peptides with moderate antimicrobial activity are: VmCT1 and VmCT2 from *V*. *mexicanus* [52]; VpAmp1.0, VpAmp1.1, VpAmp2.0 and VpAmp2.1 from *V*. *punctatus* [45]; and UyCT2 from *U*. *yaschenkoi* [51].

The effectiveness of the QnCs-BUAP peptide can be classified as weak activity (>30 μM) against *Streptococcus* (see Table 2). Other peptides that act in this concentration range are: IsCT1, IsCT2 from *Opisthacanthus madagascariensis* and its analogs [53]; and VpAmp2.0 and VpAmp2.1 from *V*. *punctatus* [45].

In this work we also assayed the synergistic effect of the consensus peptide QnCs-BUAP combined with each of the other peptides. The QnCs-BUAP peptide was chosen due to the following characteristics: a) of the four peptides, QnCs-BUAP has the highest percentage of identity with peptides Uy17 (46.15%), Uy192 (46.15%) and Uy234 (53.85%); b) according to the results of circular dichroism, the Uy234 and QnCs-BUAP peptides show a higher signal at approximately 220 nm, indicative of the formation of the α-helix structure; which is characteristic of this type of antimicrobial peptides and necessary to exert its antimicrobial activity. Of these peptides, Uy234 had very good antimicrobial activity but also the highest hemolytic activity; while the QnCs-BUAP peptide had the lowest hemolytic activity; c) IsCT-type peptides with a higher net positive charge present a higher antimicrobial activity, but also a higher hemolytic activity [53], so we selected the QnCs-BUAP peptide because when it was combined with each of the other 3 peptides, the total combined net positive charge did not surpass +4 charge, maintaining good antimicrobial activity and low hemolytic activity. QnCs-BUAP, which has a net charge +1, combined with Uy17 peptide makes a total combined charge of +3; the combination of QnCs-BUAP with Uy192 makes a total combined charge of +3; and when combined with the Uy234 peptide makes a total combined charge of +4.

*Burkholderia cepacia*, which is an unusual opportunistic pathogen that threatens immunocompromised patients [54], was resistant to all the peptides when they were tested individually, even at the highest concentrations that were evaluated. However, the combination of QnCs-BUAP with the peptides Uy234 and Uy192 displayed promising activity against *Burkholderia cepacia*, with MBCs of 150 μM and 300 μM, respectively.

The effectiveness of peptide combinations has been reported, and different advantages of their use have been proposed, such as using lower doses, cost savings, and preventing the development of resistance [55]. Combining the peptide QnCs-BUAP with the peptides Uy234, Uy17, and Uy192 resulted in the reduction of the MBC in almost all bacterial strains evaluated. Furthermore, a synergistic behavior was displayed in 25% of the resulting combinations, 37.5% displayed an additive effect, and only 8% showed antagonism.

Some AMPs from scorpion venom have shown great potential to inhibit the growth of a broad spectrum of bacteria, including MDR clinical isolates; however, their high cytotoxicity and hemolytic activity have prevented their clinical use [45,56]. According to our results, the three AMPs from *U*. *yaschenkoi* and the consensus peptide QnCs-BUAP displayed remarkably low hemolytic activity compared to that of other AMPs from scorpion venom [17,27,48,56,57]. The hemolytic concentration (HC) of the peptides Uy17, Uy192 and Uy234 towards pig erythrocytes was previously reported with an HC_25_ = 26.65 μM, 35.85 μM and 55.14 μM, respectively [28]; in our work, the same peptide sequences showed lower hemolytic activity against human erythrocytes, requiring greater peptide concentrations (> 370 μM) to achieve the same HC_25_ (see Fig 3). The different concentration at which the evaluated AMPs caused hemolytic activity (higher concentration) or bactericidal activity (low concentration) may be due to the fact that erythrocytes and microbial cell membranes have different lipid content, which influences the interaction of AMP-membranes and the mode of action. The cell membranes of mammalian erythrocytes contain zwitterionic phospholipids, such as sphingomyelin and phosphatidylcholine (PC), in addition to sterols, such as cholesterol that regulates membrane fluidity [58]. In a different way, the cytoplasmic membrane of bacteria is constituted by anionic lipids, such as phosphatidylglycerol, cardiolipin and phosphatidylserine, together with zwitterionic lipid such as phosphatidylethanolamine, which are important for the organization of the membrane [58]. Besides, a relationship between the content of peptides in the membrane and the activity of AMPs has been reported [59]; in this way, considering the size of an erythrocyte and therefore its membrane, which is larger (6–8 μm in diameter) than a bacterial cell (0.5–5 μm in length), it is expected that a higher concentration of AMP is required to affect their membrane.

Moreover, the evaluation of hemolytic activity was necessary since scorpion venom AMPs are not receptor-specific and can form pores in other cell membranes [57].

In addition, the predicted structure analysis of the helical wheel projections suggested that these peptides have a cationic α-helical structure, which was experimentally confirmed by the CD spectra. These cationic characteristics represent a peculiar structure for this type of antimicrobial peptide [17,27], which are also necessary to bind the bacterial membranes [47]. Although membrane pore formation has been proposed as the main mechanism of action of AMPs, this mechanism still needs to be confirmed for our peptides.

The mechanism of the antimicrobial action of these AMPs is not fully understood; however, it is generally accepted that the ability of AMPs to kill microorganisms depends upon their capacity to bind to the membranes of these microorganisms [60]. The ability of an AMP to interact with a target membrane depends upon its structure and the membrane target [61]; in the case of short-chain AMPs without cysteine, the α-helical structure of the AMPs is key to achieving the interaction with the membrane target, as is the higher positive charge that is provided by the amidation of the C-terminal residue [62]. The antimicrobial peptides evaluated in this article showed strong activity against *Streptococcus sp*., while exerted none effect against *Burkholderia cepacia*, it may be due to *Streptococcus sp*. is a Gram-positive bacterium, so it does not have an outer cellular membrane making it a sensitive target of the action of the AMPs. On the other hand, *Burkholderia cepacia* is a Gram-negative bacterium, so it has a double cell membrane representing a double barrier against the action of AMPs.

Furthermore, there is a relationship of bacterial membrane lipid composition and the potency of certain antimicrobial agents. Bacteria vary widely in the lipid composition (cardiolipin, phosphatidylglycerol and phosphatidylethanolamine) of their membranes and would therefore be expected to exhibit different sensitivities to antimicrobial compounds [59]. This may explain why the different Gram-Positive and Gram-negative bacteria evaluated in this work have a different degree of susceptibility to the peptides tested.

In general, Gram-negative bacteria show higher phosphatidylethanolamine content than Gram-positive bacteria. However, Gram-positive bacteria exhibit a higher amount of anionic lipids (phosphatidylglycerol and cardiolipin). The presence of charge in the lipids favors the electrostatic interactions with cationic AMPs, followed by insertion of peptides in the lipid bilayer and then the AMPs destabilize the membrane making it permeable or disrupting it [5].

Finally, the synergic effect of each peptide with the QnCs-BUAP peptide may be due to the fact that each combination results in a higher positive charge, which can achieve greater interaction with the lipids of the membrane and lead to destabilize it causing its rupture.

The IsCT and IsCT2 peptides from the venom of the African scorpion *Opisthacanthus madagascarienis* were the first peptides studied of this type of AMP [16,21]. Structure-function analysis of IsCT and IsCT2 revealed a linear α-helical structure [16,21,63]. The reduced antimicrobial activity of non-amidated AMPs is due to the decrease in the positive surface charge and the structural alteration of the α-helix, which affects the ability of the AMP to disrupt the cell membrane [64].

The estimation of secondary structure elements was determined by CD and showed that all AMPs evaluated had an α-helical structure (Fig 5), with the highest percentage of helix 1 in the peptides Uy234 and QnCs-BUAP and the highest percentage of helix 2 in the Uy234 and Uy192 peptides (Table 5).

Taking all of these results together with the positive charge values of each peptide (Table 1), the Uy234 peptide can be considered the best antimicrobial peptide, which was confirmed by the results of the antibacterial tests (Table 2).

Peptides Uy17 and Uy192 have an α-helical structure that is not as defined as that of Uy234; however, the positive charge of these peptides is greater than the charge of QnCs-BUAP, which may explain why they present higher bactericidal activity against the evaluated strains (Table 2).

When the consensus peptide was used in combination with the other *U*. *yaschenkoi* AMPs, it displayed interesting results: the best bactericidal activity was presented by the combination of the consensus peptide QnCs-BUAP and the Uy234 peptide (Table 3), since a lower MBC was achieved against 7 of the 8 bacterial strains evaluated with this combination. Furthermore, this combination of peptides achieved a bactericidal effect against *Burkholderia cepacia* (Table 3), a strain that was resistant to all peptides when they were individually evaluated (Table 2).

The highest antimicrobial activity was achieved by the combination of the Uy234 and consensus QnCs-BUAP peptides, which may be because they have the most α-helical structure and a total positive charge = + 4.

Synergism assays revealed the effectiveness of the combined use of the four peptides, although the mechanism of synergism remains to be elucidated.

The antimicrobial peptides evaluated in this work could be valuable tools for the design and development of effective AMPs against bacteria resistant to the currently available antibiotics.

## Supporting information

S1 Fig Far-UV Cd spectra of the peptide Uy192. (PDF)

S2 Fig HPLC report for Uy192. (PDF)

S3 Fig HPLC report for QnCs-BUAP. (PDF)

S4 Fig HPLC report for Uy234. (PDF)

S5 Fig HPLC report for Uy17. (PDF)

S6 Fig Uy192 mass spectrometry results. (PDF)

S7 Fig QnCs-BUAP mass spectrometry results. (PDF)

S8 Fig Uy234 mass spectrometry results. (PDF)

S9 Fig Uy17 mass spectrometry results. (PDF)
