# Supplementary figures and images for: Structural characterization of scorpion peptides and their bactericidal activity against clinical isolates of multidrug-resistant bacteria
Source: PLoS One. 2019 Nov 11;14(11):e0222438. doi: 10.1371/journal.pone.0222438 (PMC6844485; doi:10.1371/journal.pone.0222438)

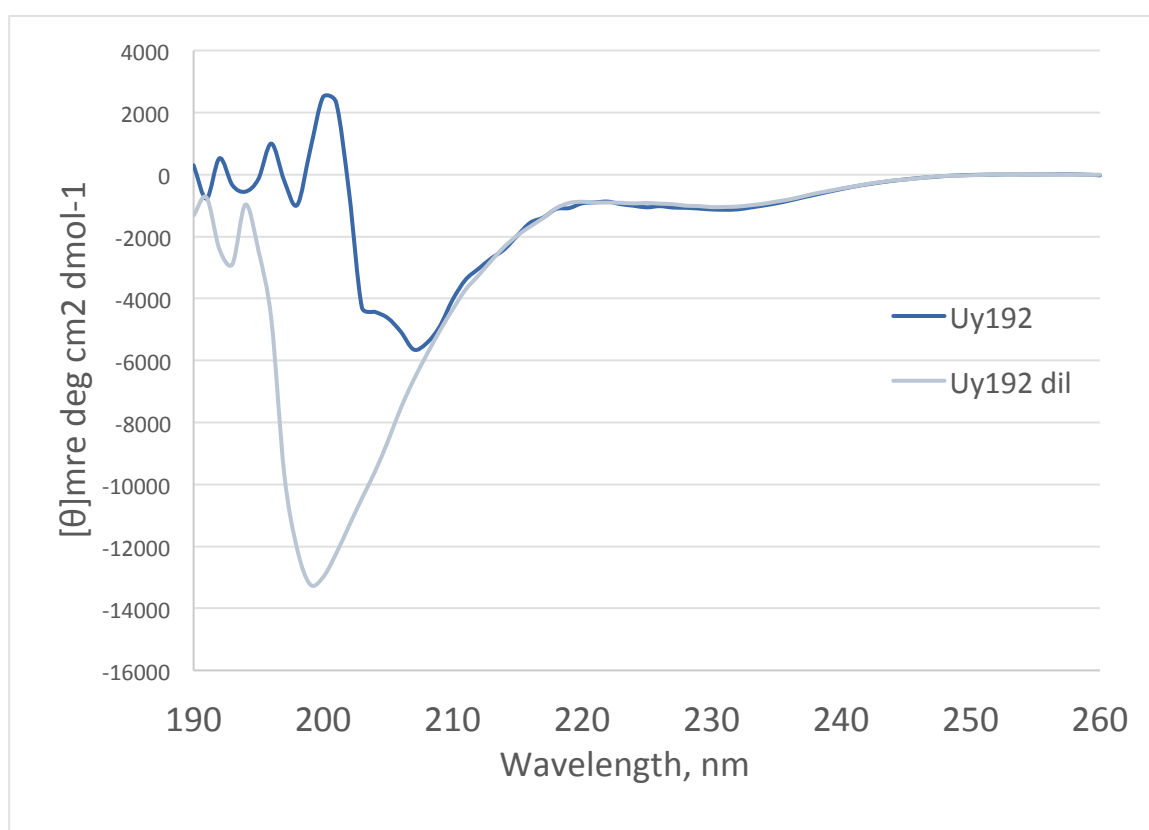

Figure S1. Far-UV Cd spectra of peptide Uy192 at 684  $\mu\text{M}$  (dark blue) and 228  $\mu\text{M}$  (light blue).

Supplement: S1 Fig — (PDF) [file pone.0222438.s001.pdf]

## Mass Spectrometry Report

12-Dec-2016

09:36:11

Peptide #2 P161202-MCX550275 MW:1369.73

161212-IL-13-NH2 62 (1.146)

Scan 60  
2.26e6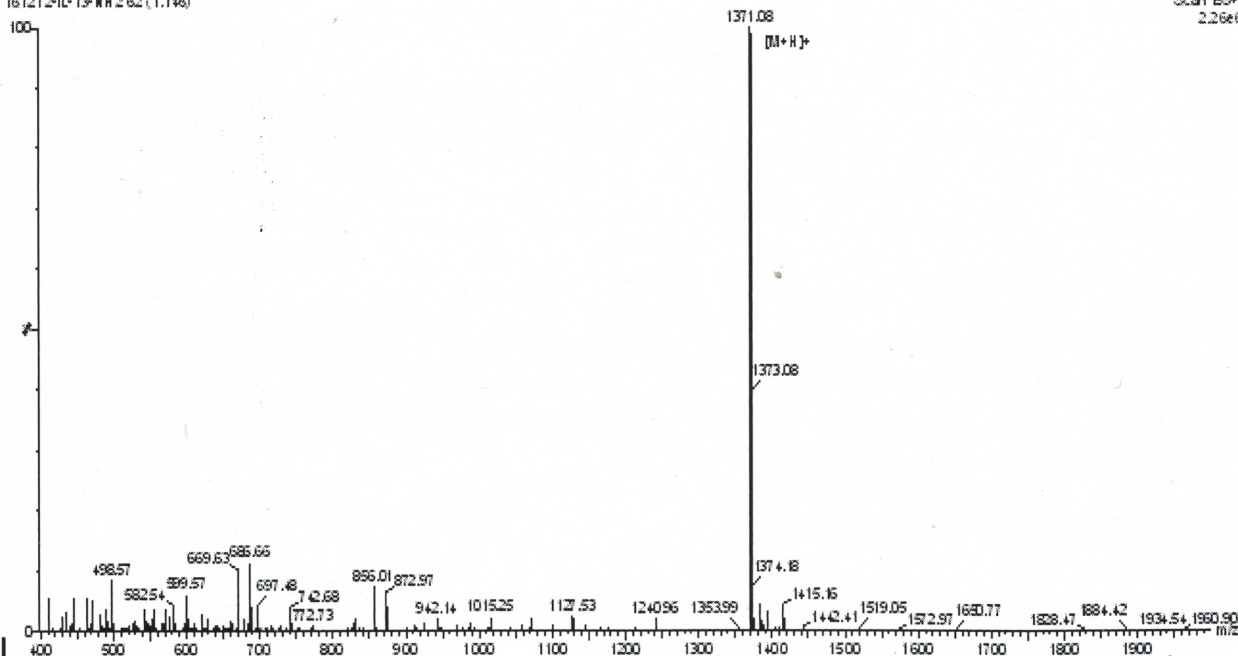

Supplement: S9 Fig — (PDF) [file pone.0222438.s009.pdf]
