# Supplementary material for: Structural characterization of scorpion peptides and their bactericidal activity against clinical isolates of multidrug-resistant bacteria
Source: PLoS One. 2019 Nov 11;14(11):e0222438. doi: 10.1371/journal.pone.0222438 (PMC6844485; doi:10.1371/journal.pone.0222438)
Supplement: S2 Fig — (PDF) [file pone.0222438.s002.pdf]

Biomatik
Tel: (519) 489-7195, (800) 836-8089
Fax: (519) 231-0140, (877) 221-3515
Email: info@biomatik.com
http://www.biomatik.com

HPLC Report

Product Name : Peptide #3 FL-13-NH2
Lot No : P170116-YS558235
Column : 4.6*250mm, Welch CX-C18 5um
Solvent A : 0.1% trifluoroacetic in 100% acetonitrile
Solvent B : 0.1% trifluoroacetic in 100% water
Gradient

	A	B
0.01min	32%	68%
25.00min	57%	43%
25.10min	100%	0%
30.0min	STOP	

Flow rate : 1.0ml/min
Wavelength : 220nm
Volume : 10ul

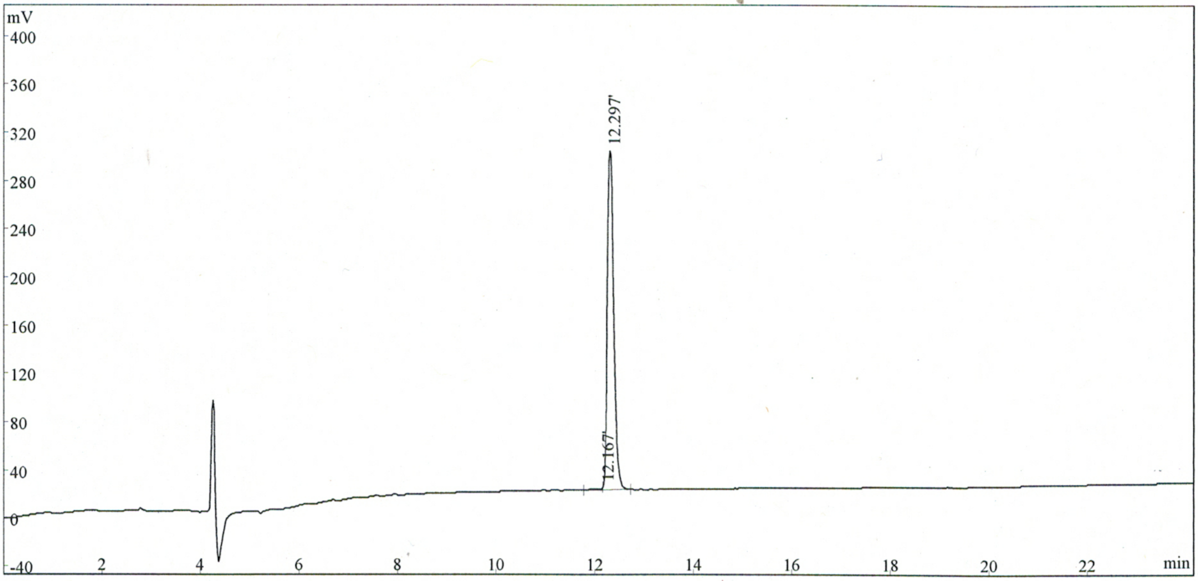

Rank	Time	Name	Conc.	Area	Height
1	12.167		0.8555	19571	2857
2	12.297		99.14	2268273	281183
Total			100	2287844	284040
