# Supplementary material for: Structural characterization of scorpion peptides and their bactericidal activity against clinical isolates of multidrug-resistant bacteria
Source: PLoS One. 2019 Nov 11;14(11):e0222438. doi: 10.1371/journal.pone.0222438 (PMC6844485; doi:10.1371/journal.pone.0222438)
Supplement: S3 Fig — (PDF) [file pone.0222438.s003.pdf]

Biomatik
Tel: (519) 489-7195, (800) 836-8089
Fax: (519) 231-0140, (877) 221-3515
Email: info@biomatik.com
http://www.biomatik.com

HPLC Report

Structure:Peptide#4 FI-13-NH2
Lot NO :P170116-YS558234
Column :250*4.6mm,Kromasil-C18-5um
Solvent A:0.1%TFA in 100%water
Solvent B:0.1%TFA in 100%acetonitrile

Gradient :	A	B
0.1min	58%	42%
25.0min	33%	67%
25.1min	0%	100%
30.0min	stop	

Flow rate:1.0ml/min
Wavelength(nm):220
Volume :10ul

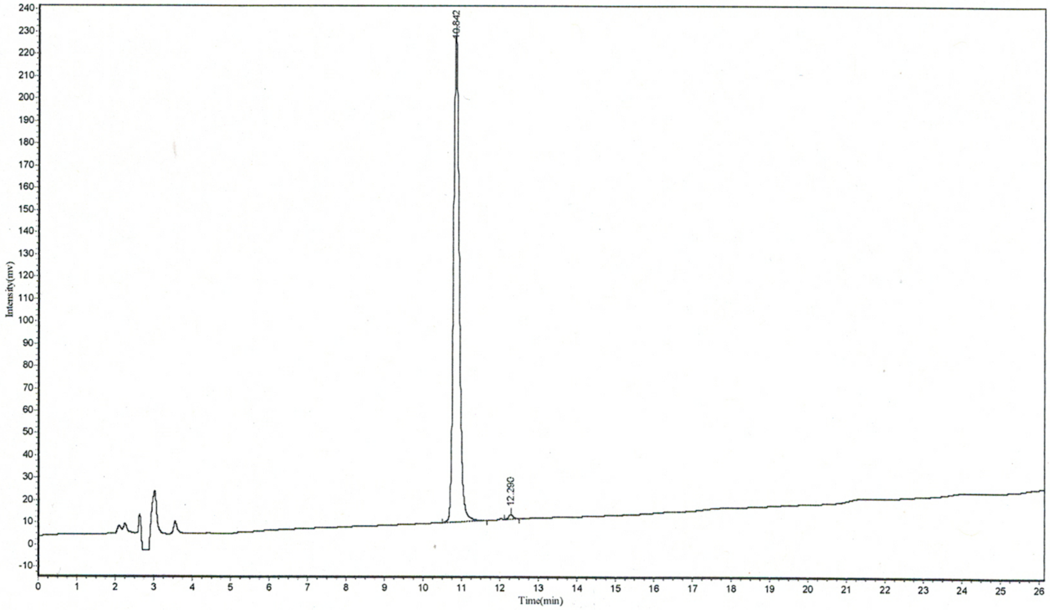

Peak No	Ret Time	Height	Area	Conc.
1	10.842	219478.453	2284248.750	99.2940
2	12.290	1940.034	16240.941	0.7060
				100.00
