# Supplementary material for: Structural characterization of scorpion peptides and their bactericidal activity against clinical isolates of multidrug-resistant bacteria
Source: PLoS One. 2019 Nov 11;14(11):e0222438. doi: 10.1371/journal.pone.0222438 (PMC6844485; doi:10.1371/journal.pone.0222438)
Supplement: S4 Fig — (PDF) [file pone.0222438.s004.pdf]

Biomatik
Tel: (519) 489-7195, (800) 836-8089
Fax: (519) 231-0140, (877) 221-3515
Email: info@biomatik.com
http://www.biomatik.com

HPLC Report

Sample Description:
Structure: Peptide #1 FL-18
Lot No:P161229-MJ550274
Column: 4.6mm*250mm, Inertsil ODS-SP
Solvent A: 0.1%Trifluoroacetic in 100% Acetonirile
Solvent B: 0.1%Trifluoroacetic in 100% Water

Gradient:	A	B
0.01min	38%	62%
25.00min	63%	37%
25.01min	100%	0%
30.00min	Stop	

Flow rate:1.0ml/min
Wavelength:220nm
Volume:10μl

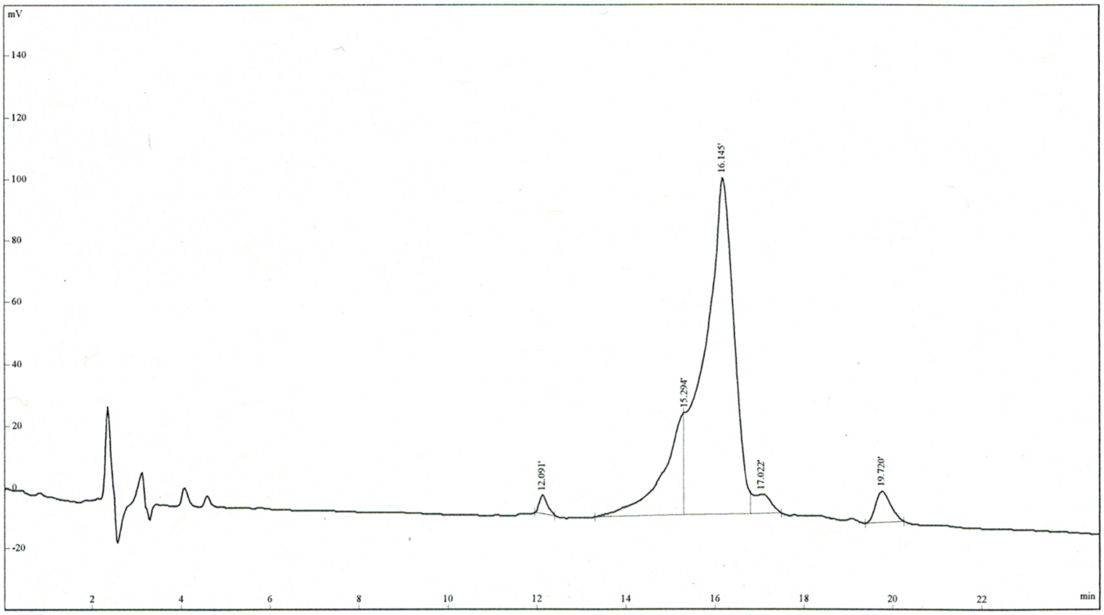

Rank	Time	Conc.	Area	Height
1	12.091	1.116	72973	5957
2	15.294	17.15	1121530	33457
3	16.145	75.44	4931986	109604
4	17.022	2.627	171727	6352
5	19.720	3.673	240146	10143
Total		100	6538362	165513
