# Supplementary material for: Structural characterization of scorpion peptides and their bactericidal activity against clinical isolates of multidrug-resistant bacteria
Source: PLoS One. 2019 Nov 11;14(11):e0222438. doi: 10.1371/journal.pone.0222438 (PMC6844485; doi:10.1371/journal.pone.0222438)
Supplement: S5 Fig — (PDF) [file pone.0222438.s005.pdf]

Biomatik
Tel: (519) 489-7195, (800) 836-8089
Fax: (519) 231-0140, (877) 221-3515
Email: info@biomatik.com
http://www.biomatik.com

HPLC Report

Product Name	Peptide #2 (IL-13-NH2)		
Lot No	P161202-MX550275		
Column	4.6mm*250mm, SinoChrom ODS-BP 5um		
Solvent A	0.1%Trifluoroacetic in 100% Acetonirile		
Solvent B	0.1%Trifluoroacetic in 100% Water		
Gradient		A	B
	0.01min	31%	69%
	25.0min	56%	44%
	25.1min	100%	0%
	30.0min	Stop	
Flow rate	1.0ml/min		
Wavelength	220nm		
Volume	10ul		

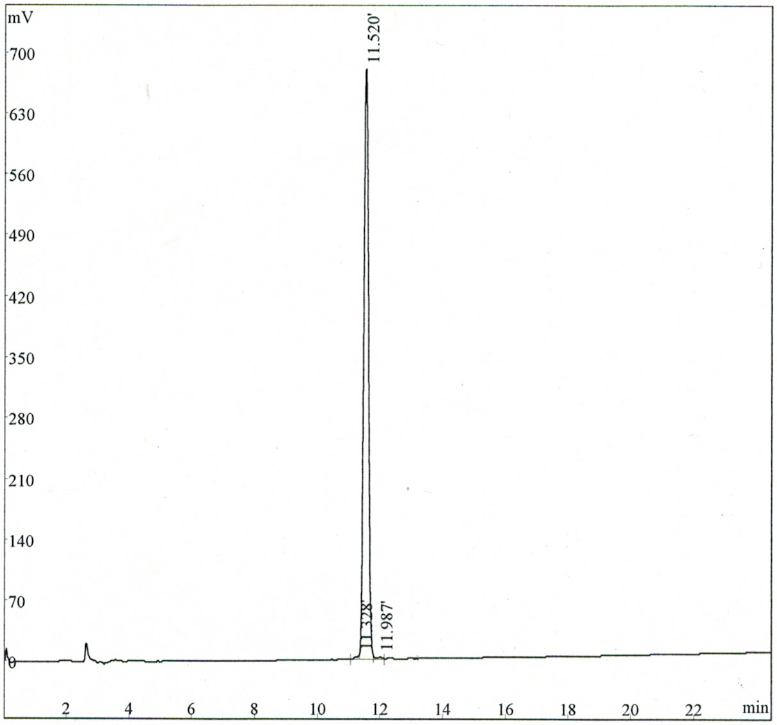

Rank	Time	Conc.	Area
1	11.328	0.5429	35453
2	11.520	99.0911	6470978
3	11.987	0.3660	23900
Total		100	6530331
