# Supplementary material for: Structural characterization of scorpion peptides and their bactericidal activity against clinical isolates of multidrug-resistant bacteria
Source: PLoS One. 2019 Nov 11;14(11):e0222438. doi: 10.1371/journal.pone.0222438 (PMC6844485; doi:10.1371/journal.pone.0222438)
Supplement: S6 Fig — (PDF) [file pone.0222438.s006.pdf]

Biomatik
Tel: (519) 489-7195, (800) 836-8089
Fax: (519) 231-0140, (877) 221-3515
Email: info@biomatik.com
http://www.biomatik.com

Mass Spectrometry Report

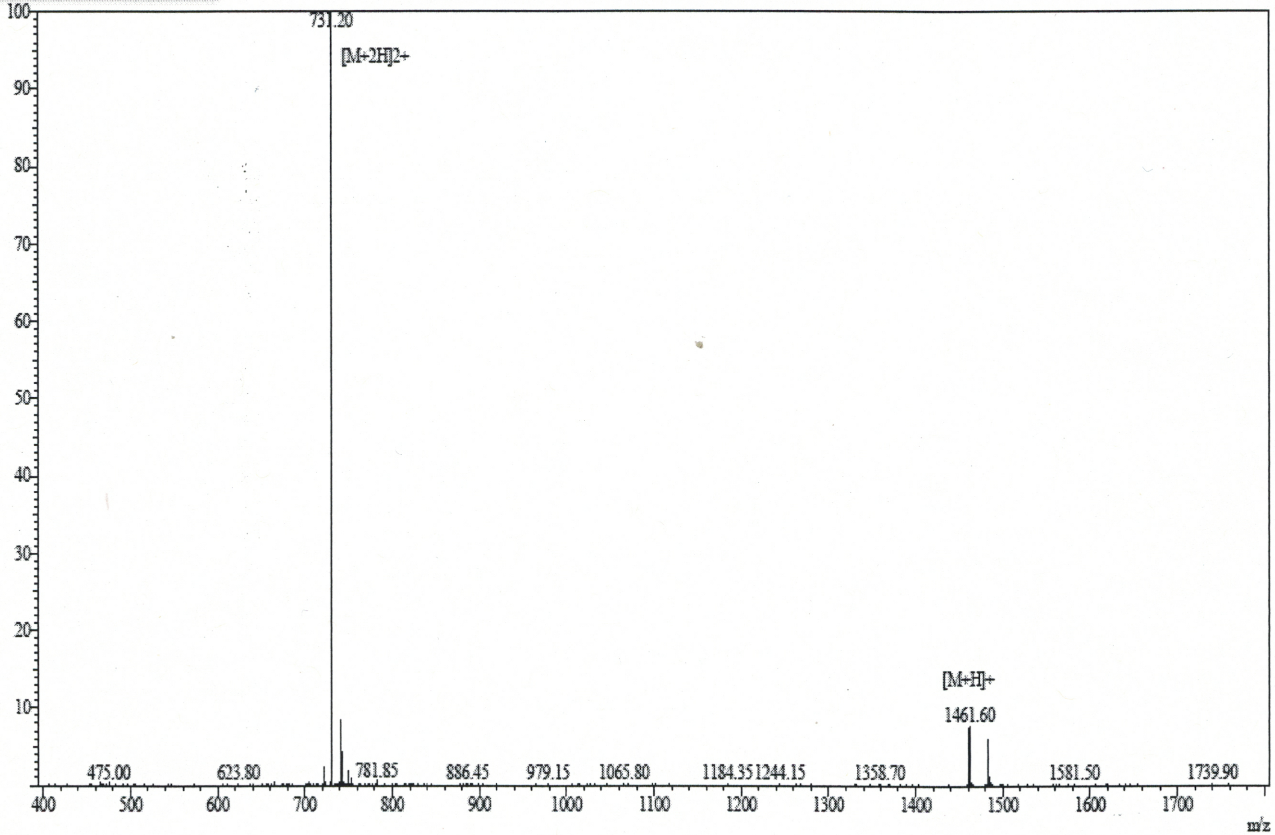

Sample Information

Date and Time	:2017-1-20
User	:Long
Product Name	: Peptide #3 FL-13-NH2
MW	:1460.80
Lot No.	:P170116-YS558235

Probe:	ESI	Probe bias:	+4.5kv
Nebulizer Gas Flow:	1.5L/min	Detector:	1.5kv
CDL:	-20.0v	T.Flow:	0.2ml/min
CDL Temp:	250℃	B.conc:	50%H2O50%ACN
Block Temp:	200℃		
