# Supplementary material for: Structural characterization of scorpion peptides and their bactericidal activity against clinical isolates of multidrug-resistant bacteria
Source: PLoS One. 2019 Nov 11;14(11):e0222438. doi: 10.1371/journal.pone.0222438 (PMC6844485; doi:10.1371/journal.pone.0222438)
Supplement: S7 Fig — (PDF) [file pone.0222438.s007.pdf]

Biomatik
Tel: (519) 489-7195, (800) 836-8089
Fax: (519) 231-0140, (877) 221-3515
Email: info@biomatik.com
http://www.biomatik.com

# Mass Spectrometry Report

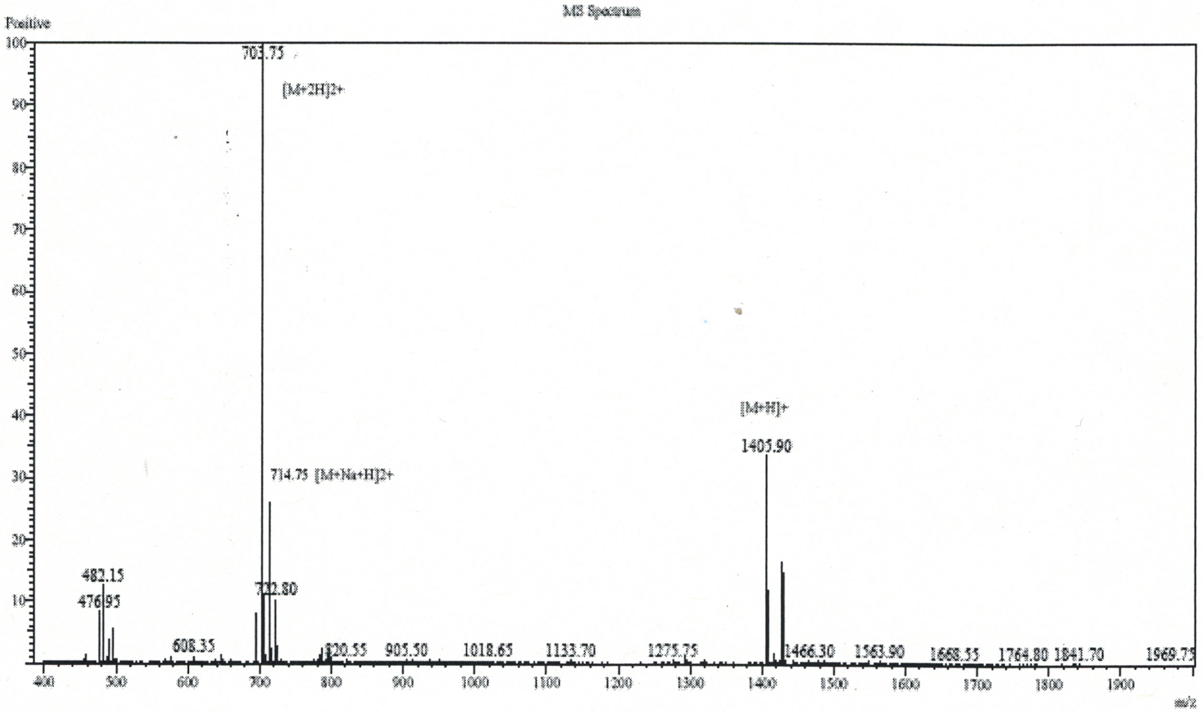


| | |
|---|---|
| Sample Information | |
| Acquired by | : Zhu |
| Time Acquired | : 15:54:37 |
| Month-Day Acquired | : 2017/2/16 |
| Injection Volume | : 1 |
| Sample Name | :Peptide#4 FI-13-NH2 |
| MW | :1405.76 |
| Lot No | :P170116-YS558234 |

<<Interface>>

| | | |
|---|---|---|
| Interface | :ESI | Probe bais :+4.5kv |
| DL Temperature | :250 C | Detector : 1.2kv |
| Nebulizing Gas Frow | :1.50 L/min | T.Flow : 0.2ml/min |
| Heat Block | :400 C | B.conc : 50%H2O/50%ACN |
| Drying Gas | :On | |
| | 10.00 L/min | |
