# Supplementary material for: Structural characterization of scorpion peptides and their bactericidal activity against clinical isolates of multidrug-resistant bacteria
Source: PLoS One. 2019 Nov 11;14(11):e0222438. doi: 10.1371/journal.pone.0222438 (PMC6844485; doi:10.1371/journal.pone.0222438)

Biomatik
Tel: (519) 489-7195, (800) 836-8089
Fax: (519) 231-0140, (877) 221-3515
Email: info@biomatik.com
http://www.biomatik.com

Mass Spectrometry Report

09-Jan-2017
12:41:11
Peptide#1 P161229-MJ550274 MW:1985.51
170109-FL-18 292 (5.397)

Probe: ESI
Cone: 50v
Desovation Temp: 350
Capillary: 3.00KV
Extractor: 5v
Gas Flow: 350
Scan ES+
2.76e7

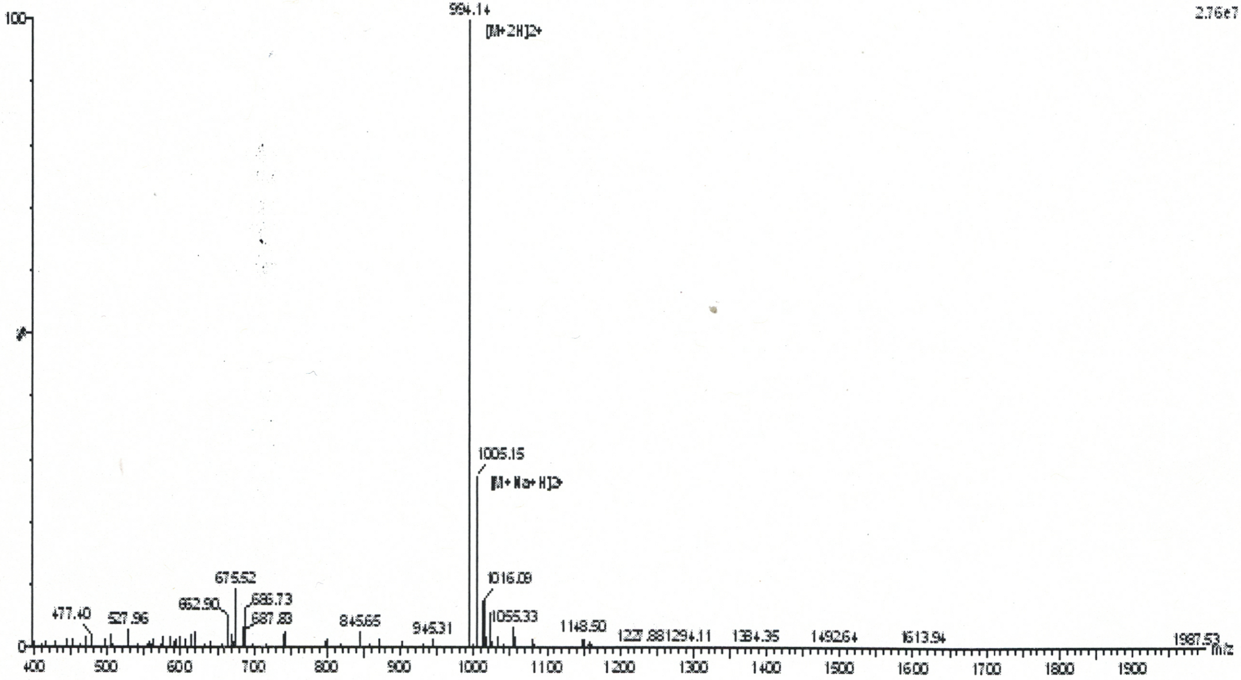

Supplement: S8 Fig — (PDF) [file pone.0222438.s008.pdf]
